# Immobilization of Methyltrioxorhenium on Mesoporous Aluminosilicate Materials

**DOI:** 10.3390/ma7042650

**Published:** 2014-03-28

**Authors:** Martina Stekrova, Radka Zdenkova, Martin Vesely, Eliska Vyskocilova, Libor Cerveny

**Affiliations:** Institute of Chemical Technology Prague, Technicka 5, 166 28 Prague 6, Czech Republic; E-Mails: radka.zdenkova@vscht.cz (R.Z.); veselyr@vscht.cz (M.V.); leitmane@vscht.cz (E.V.); libor.cerveny@vscht.cz (L.C.)

**Keywords:** methyltrioxorhenium, aluminosilicate, MCM-41, mesoporous alumina, metathesis

## Abstract

The presented report focuses on an in-depth detailed characterization of immobilized methyltrioxorhenium (MTO), giving catalysts with a wide spectra of utilization. The range of mesoporous materials with different SiO_2_/Al_2_O_3_ ratios, namely mesoporous alumina (MA), aluminosilicates type Siral (with Al content 60%–90%) and MCM-41, were used as supports for immobilization of MTO. The tested support materials (aluminous/siliceous) exhibited high surface area, well-defined regular structure and narrow pore size distribution of mesopores, and therefore represent excellent supports for the active components. Some of the supports were modified by zinc chloride in order to obtain catalysts with higher activities for instance in metathesis reactions. The immobilization of MTO was optimized using these supports and it was successful using all supports. The success of the immobilization of MTO and the properties of the prepared heterogeneous catalysts were characterized using X-ray Fluorescence (XRF), atomic absorption spectroscopy (AAS), X-ray powder diffraction (XRD), scanning electron microscopy (SEM), physical adsorption of N_2_, ultraviolet-visible spectroscopy (UV-Vis), infrared spectroscopy (FTIR), Fourier Transform Infrared Spectroscopy (FTIR) using pyridine as a probe molecule and X-ray photoelectron spectroscopy (XPS). Furthermore, the catalytic activity of the immobilized MTO on the tested supports was demonstrated on metathesis reactions of various substrates.

## 1. Introduction

Methyltrioxorhenium (MTO) was described by Hermann *et al.* more than 30 years ago as the first organometallic rhenium oxide [1–3]. Methyltrioxorhenium is a homogeneous catalyst with a wide spectrum of utilization in various organic syntheses, e.g., in oxidation reactions (epoxidation of olefins [2,4,5], Bayer-Villiger oxidation [6], oxidation of alcohols [5], oxidation of amines [7]), in Diels-Alders reactions [5,8] or in the metathesis of olefins [9,10].

The trend of using heterogeneous catalysts in chemical synthesis is motivated by a reduced environmental impact, the possibility of reuse of the catalyst or simple separation of the catalyst from the reaction mixture. In 2007 a review [11] on MTO heterogenization on different supports was carried out, and since then the amount of new data obtained in this area to date has been relatively small [12–15]. From the point of view of previous studies [16,17] in the field of MTO utilization (metathesis and olefin epoxidation), we would like to focus on the immobilization of MTO on inorganic alumina, silica and aluminosilicate supports.

The heterogenization of MTO is necessary for the successful performance of the metathesis reaction [10], since it is well known that metathesis reactions are initialized by metallocarbene present in the catalyst. MTO contains only the alkyl group, but carbene can be formed from an alkyl substituent of MTO after its immobilization [10]. Materials with Lewis acidity [18–21] such as niobium pentoxide, alumina and aluminosilicates were shown to be suitable supports. The increase of the catalytic properties was visible after modification of these materials by zinc salts [13,19]. For the immobilization of MTO used for olefin epoxidation, different approaches included the use of functionalized silicas [22–25] and numerous resins, polymers and other organic materials. In all approaches, MTO was immobilized via the Schlenk technique or by sublimation.

In this paper we provide a deep insight into the immobilization of MTO on various inorganic supports, namely MCM-41, mesoporous aluminas and aluminosilicates (type Siral). All these materials exhibit a high surface area, well-defined regular architecture and narrow pore size distribution of mesopores and therefore represent excellent supports for the active components. The chosen materials are moreover modified by zinc chloride in order to obtain a more active complex system. A comparison of their loading ability and broad characterization is given. These catalysts have possible applications in olefin metathesis and in olefin epoxidation. The catalytic activity of the immobilized MTO on various supports is demonstrated on the various metathesis reactions in the presented paper.

## 2. Results and Discussion

Five different aluminosilicate supports of type Siral (Siral 10, Siral 20, Siral 30, Siral 30HP and Siral 40) with different Al_2_O_3_/SiO_2_ ratio, pore volume, surface area and bulk density were tested for immobilization of MTO. Siral 30HP is characterized by wide pore diameter and higher pore volume in comparison to other supports from the Siral series. Pore volumes of Siral 10, 20, 30 and 40 are within the 0.75–0.9 mL/g range, but Siral 30HP is characterized by a pore volume of 1.4 mL/g according to the supplier’s datasheet.

The presence of mesoporous molecular sieve MCM-41 and mesoporous alumina (MA 5) were also tested.

### 2.1. Catalysts Characterization

#### 2.1.1. Quantitative Studies by X-ray Fluorescence (XRF) and Atomic Absorption Spectroscopy (AAS)

The amount of supported MTO on various materials was detected by XRF and AAS methods. Results of the two methods differed from one another by about 20%–30%, which is due to differences in their sensitivity and reliability. The results of AAS indicated a lower amount of immobilized MTO because of incomplete leaching of MTO from support using 1 M hydrochloric acid. On the other hand, the order of amount of immobilized MTO on supports was not changed. The following data were calculated mainly from results obtained by the XRF method.

Optimization of the immobilization of MTO was performed. Four different anhydrous solvents were used for the immobilization procedure, namely dichloromethane, trichloromethane, toluene and ethanol. The highest immobilized amount of MTO was achieved using dichloromethane for all supports. Ethanol as a solvent for immobilization could be in some cases very inconvenient due to its competition with the immobilized complex. The immobilization experiments were performed using dichloromethane at different temperatures and with different times. The highest amounts of immobilized MTO after 20 h were achieved at room temperature, however the amount of immobilized MTO was generally not significantly influenced by temperature and the observed differences did not exceed 6%.

Table 1 shows the immobilized amount of MTO on supports (calculated from XRF data, which was also confirmed by AAS) for two different nominal amounts of MTO (5 and 15 mg of MTO on 100 mg of the support). The loaded amount of MTO per 100 mg of support was calculated.

The highest immobilized amount of MTO was achieved using Siral 40, Siral 20 and MCM-41 as supports, amounting to over 14 mg of MTO on 100 mg of support in the case of nominal loading of 15 mg MTO/100 mg of support and around 4.5 mg of MTO on 100 mg of support in the case of nominal loading of 5 mg MTO/100 mg of support. The success of the immobilization was then 94%–97% in case of the higher nominal loading of MTO and 88%–90% in case of the lower nominal loading of MTO.

The immobilization success for the Siral supports seems to increase with increasing surface area of the used supports. Siral 30 however falls outside of this trend; this observation was not explained.

The lowest immobilized amount of MTO (33%) was achieved using Siral 30HP as a support which is characterized by wide pore diameter and higher pore volume in comparison to other supports from the Siral series.

The immobilized amounts of MTO on supports modified by zinc chloride are also shown in Table 1. The success of modification of zinc chloride was around 80% using all supports. It is obvious that this modification lead to a 10% increase of immobilized amount of MTO on MA 5 in case of immobilization of 5 mg MTO/100 mg of MA. On the other hand, 8% and 30%–50% decreases of immobilized amounts of MTO on modified Siral materials were observed after support modification by zinc chloride in case of immobilization of 5 and 15 mg MTO/100 mg Sirals, respectively. The ratios of Zn:Cl were determined for these samples by the XRF method because of the different behavior of Siral supports and mesoporous alumina caused by zinc chloride modification. In case of Siral supports, the ratio of Zn:Cl was 1:0.9 in both cases (Siral 20 and 40). This means that some Zn atoms are present on the support without a bond to Cl and therefore they probably form a bond to oxygen atoms of supports. This leads to the conclusion that the amount of potential sites for MTO immobilization decreases in comparison to a pure support. This fact has been already published by Tovar *et al.* and the possibility of a Re-O_support_ bond formation has been also suggested [13]. On the other hand, the ratio of Zn:Cl was 1:1.6 in case of zinc chloride modification of mesoporous alumina, which is close to the stoichiometric ratio (1:2). Tovar *et al.* have also described the possible formation of a Re-Cl bond. This fact can explain the increased success rate of MTO immobilization on ZnCl_2_/MA 5.

Table 2 shows the immobilized amount of MTO on selected supports (calculated from XRF data) for a nominal amount of 30 mg of MTO on 100 mg of the support. These data confirm that the sorption capacities of the supports are high enough and therefore they are also able to bind higher amounts of catalysts. The success rate of the immobilization of 30 mg of MTO was comparable to the success rate for 15 mg of MTO.

#### 2.1.2. Morphological Studies by Scanning Electron Microscopy (SEM)

The morphology of the prepared supports of their zinc chloride modified form and of the catalytic system with immobilized MTO (15 mg MTO/100 mg of the support and 5 mg MTO/ZnCl_2_/support in case of using zinc modified support) was studied by scanning electron microscopy (see Supplementary Information, Figures S1–S3).

The support materials were characterized by different particle sizes. Prepared MCM-41 had small particles (<1 μm) which form large agglomerates (Figure S1). No apparent changes in crystal morphology were observed after immobilization of MTO on MCM-41. Particle size and agglomerates remained the same (Figure S1).

The prepared mesoporous alumina (MA 5) is quite heterogeneous in particle size (Figure S2). The smallest particles are smaller than 10 μm and form agglomerates of sizes of about 40 μm. No apparent changes in crystal morphology were observed after modification of the MA (Figure S2) and Siral 20 and Siral 40 (Figure S3) supports by zinc chloride.

Immobilized MTO was not visible by scanning electron microscopy on pure supports, and the same also applies to their zinc-modified form (see Supplementary Information, Figures S1–S3).

The elemental maps of chlorine, rhenium and zinc on immobilized MTO on ZnCl_2_/Siral 40 were achieved using EDS. Elemental maps revealed homogeneous distribution of zinc, chlorine and rhenium on the sample. Image processing in the MATLAB environment was used to quantify discrepancies in the elemental maps for each element. Colored images were converted to grayscale images, which were subsequently segmented to a binary image with a threshold value equal to zero. Then each binary image was represented by a matrix. We subtracted the matrices of zinc, chlorine and rhenium, and the remainder after subtraction characterized the discrepancies between elemental maps. The presence of zinc, chlorine and rhenium differed only in 974 pixels from the total number of 3,8967 pixels. Therefore we assumed that zinc, chlorine and rhenium were located in the same positions on the support.

#### 2.1.3. Results of Nitrogen Adsorption Measurements

The specific surface areas, the pore volumes and the average pore diameters of supports and of immobilized MTO on them determined by nitrogen adsorption are summarized in Table 3. The specific surface areas were calculated by the Brunauer-Emmett-Teller (BET) method and the average pore diameters were obtained based on the Barrett-Joiner-Halenda (BJH) correlation from adsorption isotherms.

The highest BET surface area was measured for MCM-41 being 1057 m^2^/g. This material is also characterized by the highest pore volume and small pore diameter.

The immobilization of MTO on the supports caused a decrease of the specific surface areas in all cases. The immobilization of catalysts on the supports caused the decrease of pore diameters and of pore volumes in all cases except for mesoporous alumina. In this case, the parameters remained the same. The decreases in measured parameters were the highest in the case of using Siral 10 as a support, amounting to a 13% difference in surface area, 9% difference in pore diameter and 24% decrease of pore volume. The decreases of these values were comparable for the use of Siral 20, Siral 40 and MCM-41, amounting to 7%–9% for surface area, 3%–4% for pore diameter and 10%–14% for pore volume.

A comparison of the results of nitrogen adsorption measurements of the native supports and those modified by zinc chloride and MTO is provided in the following table (Table 4). The modification of the supports by zinc chloride caused a significant decrease of specific surface area and of pore volume. On the other hand, changes in pore diameters were not observed after modification by ZnCl_2_. This could be explained by blocking of the entrances to pores by the ZnCl_2_ molecule bonded on the support. No significant changes in specific surface area, pore diameter and pore volume after loading MTO on ZnCl_2_ modified MA 5 were observed. On the other hand, the decrease of surface area without any changes in pore diameter was observed after immobilization of MTO on ZnCl_2_/Siral materials.

**Figure 1.**
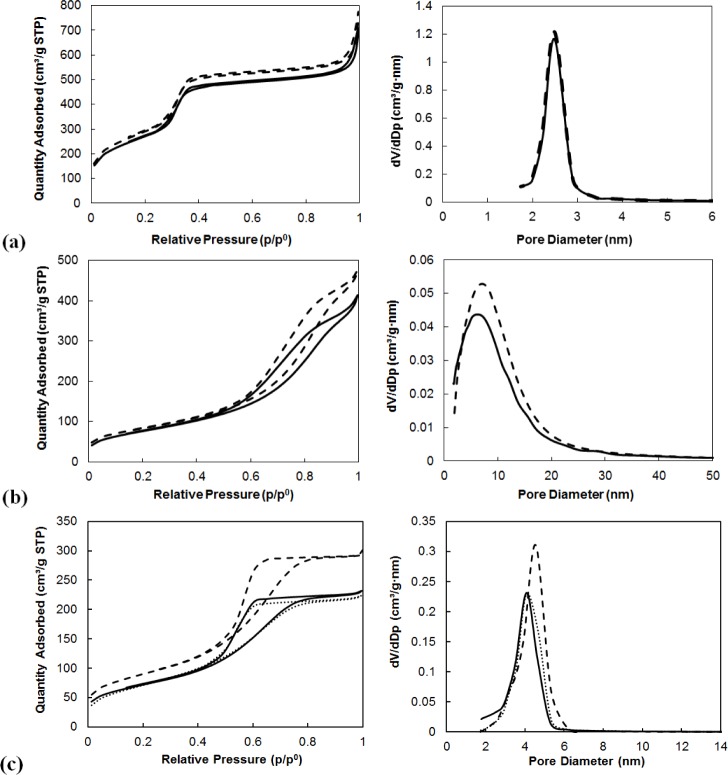
Adsorption and desorption isotherms and pore size distribution of (**a**) MCM-41 (dashed line) and MTO/MCM-41 (line); (**b**) Siral 20 (dashed line) and MTO/Siral 20 (line) and (**c**) MA 5 (dashed line), ZnCl_2_/MA 5 (dots) and MTO/ZnCl_2_/MA5 (line), (immobilized amount was 15 mg methyltrioxorhenium (MTO) on 100 mg of pure supports and 5 mg MTO on ZnCl_2_/support).

The adsorption and desorption isotherms of N_2_ for the native supports are compared with the isotherms for zinc chloride modified supports and supports with immobilized MTO (Figure 1).

Adsorption isotherms and Tables 3 and 4 show that the pore diameter is just slightly reduced by binding of MTO or by zinc chloride modification of supports.

#### 2.1.4. Structural Analysis of the Catalysts by X-ray Powder Diffractometer

**Figure 2.**
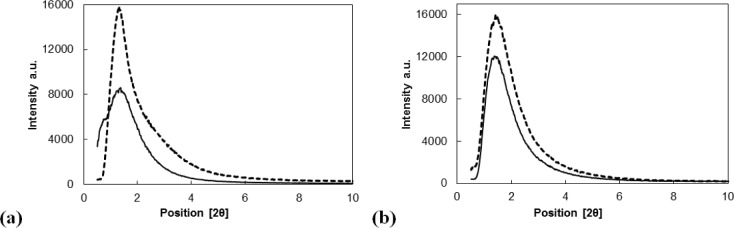
Diffractograms (**a**) of Siral 40 (line) and MTO/Siral 40 (dashed line) (15 mg MTO/100 mg Siral 40) and (**b**) of mesoporous alumina MA 5 (line) and MTO/ZnCl_2_/MA 5 (dashed line) (5 mg MTO/100 mg ZnCl_2_/MA 5).

The structures of all tested materials were verified by X-ray powder diffraction. Figure 2a,b show the diffractograms of Siral 40 and MTO/Siral 40 (Figure 2a) and of mesoporous alumina (MA 5) and MTO/ZnCl_2_/MA 5 (Figure 2b). The MTO peaks were observed neither in the MTO-modified catalysts nor in their ZnCl_2_ modified forms, pointing to a perfect dispersion of MTO particles on the support materials.

Figures depict the diffraction patterns of the supports and of MTO modified supports at low angles. A significant shift of the basal peak was not observed. This confirms the fact that the pore size remained the same and the mesoporous structure was preserved. This conclusion is consistent with the results obtained using physical adsorption of nitrogen.

#### 2.1.5. Ultraviolet-Visible Spectroscopy (UV-Vis)

**Figure 3.**
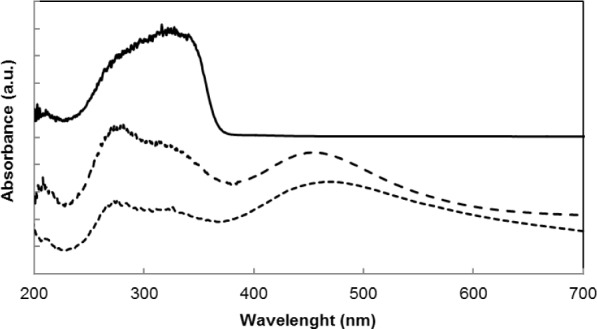
Solid state ultraviolet-visible spectroscopy (UV/VIS) spectra of pure MTO (line) and MTO immobilized on Siral 40 (dashed line) and on ZnCl_2_/MA 5 (dots).

The solid-state UV-Vis spectra were measured for pure MTO, native supports and supports modified by ZnCl_2_. The obtained spectra were compared (Figure 3). A band around 470 nm was observed in the samples modified by MTO. This band indicated a d^8^–d^8^ interaction of two Re atoms [26]. This was explained by the formation of dimers of MTO on the surface of the supports (Figure 7). The dimers of MTO were formed on all native supports and on their zinc chloride modified forms. The formation of dimers has been described for Grubbs-type Ru catalysts [27]. The possibility of MTO forming dimers has been previously studied in [28].

**Figure 4.**
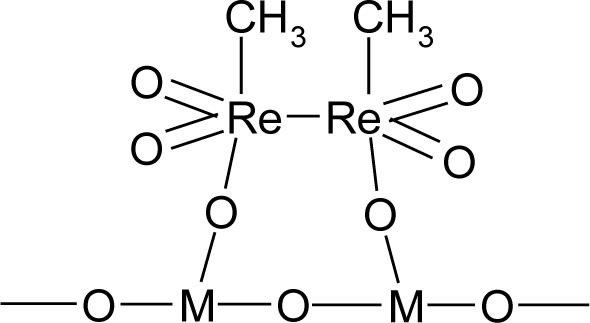
Structure of MTO dimer based on data obtained from UV-Vis spectra (M = Si, Al).

The structure of MTO dimer bound on the support surface is depicted in Figure 4. Rhenium is presented in this molecule in the oxidation state +VI.

#### 2.1.6. Fourier Transform Infrared Spectroscopy (FTIR)

The structural characterization of MTO binding was performed by infrared spectroscopy using FTIR. FTIR spectra were measured for the supports Siral 20, Siral 40, MCM-41 and ZnCl_2_/MA 5 and were compared with the spectra of MTO modified supports. The spectrum of pure MTO showed various bands. Symmetric and asymmetric vibrations of the Re = O valence group are present in the 915–1000 cm^−1^ band [29]. The band around 575 cm^−1^ is assigned to the Re-C group vibration [29]. The bands around 2984, 2898 and 1359 cm^−1^ are assigned to stretching vibrations of the –CH_3_ group.

**Figure 5.**
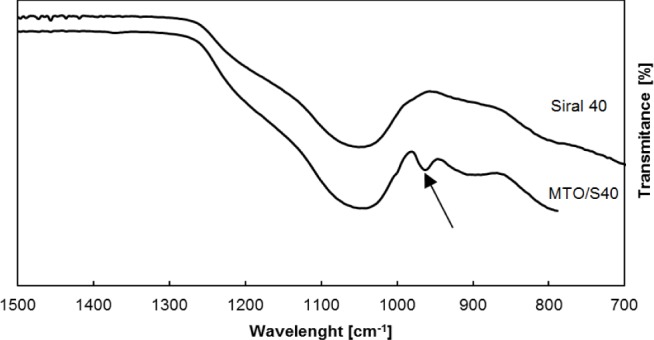
Fourier transform infrared spectroscopy (FTIR) spectrum of Siral 40 and its MTO modified form (15 mg MTO/100 mg Siral 40).

Comparing spectra of Siral 40 and MTO/Siral 40 (Figure 5) shows a band around 960 cm^−1^, which indicates the presence of an asymmetric vibration of Re = O (marked with an arrow). The band around 960 cm^−1^ was observed also in the samples MTO/MCM-41, MTO/Siral 20 and MTO/ZnCl_2_/MA 5. The bands 1206, 1060 and 794 cm^−1^ are assigned to the stretching vibration of mesoporous silicate material (Si-O-Si) [29]. Symmetric vibration of Re = O is masked by the strong vibration of Si-O-Si.

#### 2.1.7. Acid Site Concentrations of the Catalysts Measured by Pyridine Adsorption/Desorption with FTIR

Concentrations of Brønsted and Lewis acid sites on the materials were determined by FTIR using pyridine as a probe molecule.

The band around 1450 cm^−1^, corresponding to the vibration of pyridine, was used to determine the Lewis acid sites. The band around 1545 cm^−1^, corresponding to the vibration of pyridinium cations, was used to determine Brønsted acid sites. The peak areas were used for the quantitative determination of the concentrations of these centers [30].

**Figure 6.**
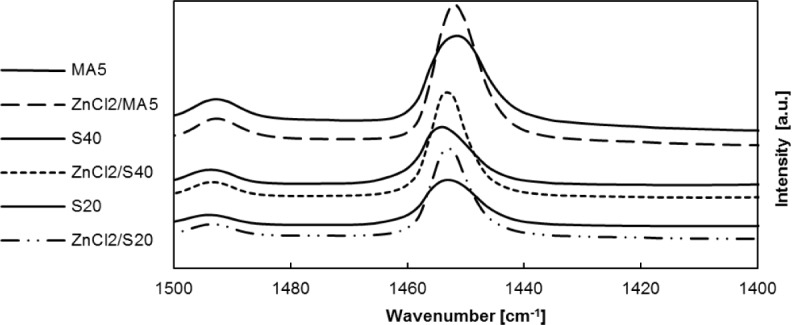
Comparison of Lewis acidity of the materials MA 5, Siral 40, Siral 20 and their ZnCl_2_ modified forms (5 mg MTO/100 mg support).

The highest concentration of Lewis acid sites was determined for pure alumina (MA 5), while Siral 40 and Siral 20 were less acidic. The modification of tested supports by ZnCl_2_ caused significant changes especially in Lewis acidity. Figure 6 compares the spectra of the native supports before and after their modification by ZnCl_2_. The Lewis acidity of the supports increased due to their modification by ZnCl_2_ in all cases. This increase was the highest on MA 5 and decreased in the following order: MA 5>Siral 20>Siral 40. It can be concluded that the higher the content of Al_2_O_3_ in the support, the higher the increase of Lewis acidity caused by ZnCl_2_ modification.

The significant changes in acidity, both Lewis and Brønsted, were observed after immobilization of MTO. Increases of the concentration of Brønsted acid sites were observed after immobilization of MTO on native Siral materials (Siral 20 and Siral 40) and on MCM-41. On the other hand, Brønsted acid sites almost disappeared due to the immobilization of MTO on ZnCl_2_/MA5. The occurrence of weak Lewis acid sites increased after immobilization of MTO on ZnCl_2_/MA5, but the occurrence of medium and strong Lewis acid sites decreased slightly. Lewis acidity increased after modification of Siral 20 and MCM-41 by MTO but it decreased after modification of Siral 40.

#### 2.1.8. X-ray Photoelectron Spectroscopy (XPS) Results

X-ray photoelectron spectroscopy of the studied catalyst samples was carried out. Three measurements were made for each sample, the first one after 3 h under vacuum, the second after 24 h under vacuum and the third one after sputtering by Ar ions with energy of 5 keV for 10 min The XPS of MTO modified supports demonstrated that rhenium is present in three oxidation states: Re^7+^, Re^6+^ and Re^4+^. The 7+ and 6+ Re atoms are known to be active species in metathesis reactions [31]. The number of oxidation states of Re is shown in Table 5.

The presence of Re^6+^ confirms the formation of MTO dimer, which was suggested based on data from UV-Vis spectroscopy. A higher amount of Re^6+^ after 3 h in vacuum was determined for the MTO/ZnCl_2_/MA 5 sample compared to the MTO/Siral 40 sample. However, the same amount of this oxidation state of Re was observed in both samples after being exposed to vacuum for 24 h—in both cases resulting in an occurrence of 41%. The occurrence of Re^7+^ increased in the MTO/ZnCl_2_/MA 5 sample after long exposition to vacuum but decreased in the MTO/Siral 40 sample. A very small amount of metallic rhenium was observed in MTO/Siral 40 catalysts after 24 h in vacuum; this could be caused by a slight decomposition of MTO. A higher amount of the 6+ atoms on the ZnCl_2_/MA 5 sample in comparison with the Siral 40 sample (after 3 h in vacuum) probably indicates a different anchoring type on the support.

The last measurement was performed for samples after sputtering by Ar ions. Data obtained from this measurement were collected at a particle depth of 20 nm. Neither Re nor Zn was observed in the sample after sputtering.

**Figure 7.**
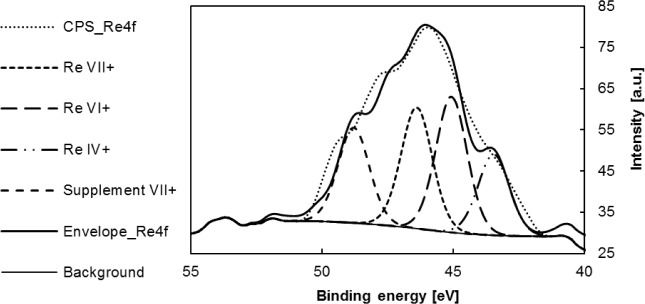
X-ray photoelectron spectroscopy (XPS) spectrum with fitted peaks of MTO/ZnCl_2_/MA5 (after 3h in vacuum).

The XPS spectrum with fitted peaks of Re oxidation states in MTO/ZnCl_2_/MA5 sample (after 3 h in vacuum) is depicted in Figure 7.

### 2.2. Results from Metathesis Reactions

As mentioned in the introduction, homogeneous MTO is inactive in metathesis reactions [10]. Therefore, the heterogenization of this catalyst is necessary for these reactions. MTO immobilized on materials with different physico-chemical properties were tested for various metathesis reactions in the presented research. It has been reported that the active Re species for metathesis reactions are Re^6+^ and Re^7+^ [31]. These Re species were also present in the tested materials according to XPS results.

Table 6 shows the results achieved in the metathesis of model compounds, namely homometathesis of methyl undec-10-enoate and of 4-allylanisole and ring-closing metathesis of diethyl diallylmalonate.

MTO/ZnCl_2_/MA 5 were evaluated as the most active catalysts in all cases. Total conversions of substrates were obtained over this catalytic complex in metathesis of methyl undec-10-enoate and diethyl diallylmalonate, with the achieved selectivities to desired products being 99% and 100%, respectively. The side products of the reactions were identified as the products of the follow-up isomerization reactions (products with different positions of the double bond).

The positive influence of ZnCl_2_ modification is significant in metathesis of methyl undec-10-enoate also when using Siral supports. MTO immobilized on Siral 20 was inactive in metathesis of this substrate and only moderate conversion was achieved by the use of MTO/Siral 40. On the other hand, the conversions of methyl undec-10-enoate were 49% and 80% over MTO/ZnCl_2_/Siral 20 and MTO/ZnCl_2_/Siral 40, respectively. Furthermore, the positive influence of support modification by ZnCl_2_ on the selectivity is evident from Table 6.

On the other hand, the catalyst: substrate ratio in our research was higher (1:17, *TON* = 17) in comparison to previously published results (*TON* >3000) [31].

Any leaching of MTO from the supports was not observed during the reactions. Rhenium contents on the supports were determined by XRF measurement for used catalysts and the achieved results were compared with Re contents in the fresh prepared catalytic systems. The data showed the same amount (or within the measurement errors) of rhenium in samples before and after the metathesis reactions.

Furthermore, MTO leaching from the supports in various media was measured for the prepared catalytic systems. The chosen tested leaching media were dichloromethane (p.a. and extra dry) and water. The results showed that the most significant leaching of MTO from the supports was observed in the presence of water. An amount of 90% of MTO was leached from Siral 40 and almost all MTO was leached from MCM-41 in water. A loss of MTO of around 50% was determined for both supports in dichloromethane (p.a.). On the other hand, very low leaching was observed in the case of using anhydrous dichloromethane as a leaching medium. It can be concluded that the presence of trace amounts of water has a negative influence on the stability of prepared catalyst complexes. This fact corresponds with previously published results by Kühn *et al.* who reported that MTO hydrolyzes rapidly in basic and slower in acidic aqueous solutions [32]. The water-soluble MTO exchanges its oxo ligands quickly with water and it has been assumed that it possibly forms octahedral water adducts prior to undergoing aggregation to polymeric MTO [33].

## 3. Experimental Section

### 3.1. Synthesis and Modification of Supporting Materials

Methyltrioxorhenium was purchased from Süd-Chemie, the aluminosilicates Siral 10, 20, 30, 30HP and 40 were purchased from Sasol and used as obtained.

MCM-41 was prepared by basic hydrothermal synthesis (based on [34]) by adding 2.4 g of cetyltrimethylammonium bromide (C_16_TMABr, Sigma-Aldrich) to 120 mL of distilled water. After 5 min, 10 mL of a water solution of NH_3_ (26 wt%, Lachema) and 10 mL of tetraethyl orthosilicate (TEOS, Sigma-Aldrich) were added. The suspension was continuously stirred at room temperature for 12 h. The formed solid was repeatedly washed by ethanol and distilled water to eliminate residual NH_3_ and alkoxides. Prepared materials were dried at 100 °C for 1 h and thermally treated at 550 °C under a N_2_ atmosphere for 2 h and then under air for 20 h. In order to improve the ability to bind the catalyst by increasing the amount of hydroxyl groups, the final MCM-41 was stirred in distilled water for 1 day, then filtered and dried at 200 °C.

Mesoporous alumina was prepared according to the literature [35]. Typically 8.4 g of aluminiumisopropoxide (Sigma-Aldrich) and 7.4 g of glucose (Lachema, Czech Republic) as a templating agent were dissolved in 100 mL of distilled water and the solution was stirred at room temperature for 30 min. Then the pH of the solution was adjusted to the desired value of pH 5 by the addition of an aqueous solution (10 wt%) of nitric acid (Penta, Czech Republic, p.a.). After letting the solution stand for 5 h, the mixture was heated and kept at 100 °C overnight in order to evaporate water and other volatiles. The solid material was heat treated at 550 °C in a stream of N_2_ for 2 h and then calcined in a stream of air at the same temperature for 24 h.

Modification of mesoporous Al_2_O_3_ and Siral materials by ZnCl_2_ (Lach-Ner, Czech Republic) was performed according to the literature [19]. Zinc chloride (0.334 g) was dissolved in ethanol and added dropwise to 2 g of the supporting materials . The mixture was stirred and dried under a N_2_ flow at 70 °C in a Schlenk flask. The material was then washed twice by ethanol and dried. The modified supporting materials were heat treated in an air flow at a temperature of 400 °C (ramp 9.6 °C/min) for 4 h.

### 3.2. Immobilization of MTO

The activation of supporting materials was performed before immobilization of MTO as follows. The supporting materials were calcined under a N_2_ atmosphere at 550 °C for 16 h and then under reduced pressure (0.8 kPa) at 120 °C for 4 h. Supporting materials modified by ZnCl_2_ were activated at 500 °C for 2 h under reduced pressure (0.8 kPa). The treated materials (1 g) were transferred into a Schlenk flask.

In a typical experiment, the flask with the support was filled with an argon atmosphere and 15 mL of anhydrous solvent (dichloromethane, trichloromethane, toluene or ethanol; Sigma-Aldrich) was added. The immobilization of MTO was performed by adding 50–300 mg (at a ratio of 5, 15 and 30 mg of MTO/100 mg of support) of the catalyst under an Argon atmosphere and the suspension was stirred for 20 h. The solvent was then removed by decantation and the solid part was dried under reduced pressure.

### 3.3. Catalysts Characterization

#### 3.3.1. X-ray Fluorescence (XRF)

The loaded amount of MTO on the supports was determined using X-ray fluorescence. An ARL 9400 XP sequential WD-XRF spectrometer (Thermo ARL, Ecublens, Switzerland) was used. The spectrometer was equipped with a Rh anode end-window X-ray tube of type 4GN fitted with a 75 μm Be window. All peak intensity data were collected by the WinXRF software tool in vacuum. The generating settings-collimator-crystal-detector combinations were optimized for all 79 measured elements with an analysis time of 6 s per element. The obtained data were evaluated by the Uniquant 4 software tool.

#### 3.3.2. Atomic Absorption Spectroscopy (AAS)

Another method used for the determination of the loaded amount of MTO on the supports was atomic absorption spectroscopy (AAS). The Spectra AA880 spectrometer with the flame technique of atomization was used. Rhenium was leached from the catalyst sample (100 mg) three times using 4 mL of 1 M hydrochloric acid. The exact volume of the solution of 1 M HCl with leached rhenium was analyzed.

#### 3.3.3. Scanning Electron Microscope (SEM)

The samples were characterized with scanning electron microscopy (SEM, Tescan Lyra3GMU/EDS/EBSD/STEM/TOFSIMS, TESCAN Brno, s.r.o., Brno, Czech Republic). The electron microscope was used to determine the crystal morphology of the pure supports as well as after ZnCl_2_ and MTO modifications.

To confirm MTO binding on the support, energy dispersive X-ray spectroscopy (EDS, Oxford-Instruments, Abingdon, United Kingdom) was used to find the elemental maps of the samples. Elemental maps were created in the Aztec software package.

#### 3.3.4. X-ray Powder Diffraction (XRD)

X-ray powder diffraction was used to confirm the crystallinity of the prepared supports (MCM-41 and alumina) and to confirm the presence of supported MTO. X-ray powder diffraction data were collected at room temperature with an X’Pert PRO θ-θ powder diffractometer with parafocusing Bragg-Brentano geometry using CuK_α_ radiation (λ = 1.5418 Å, U = 40 kV, I = 30 mA). Data were scanned with an X’Celerator ultrafast detector over the angular range of 5°–64° (2θ) with a step size of 0.0167° (2θ) and a counting time of 20.32 s step^−1^. Data evaluation was carried out in the HighScore Plus software package.

#### 3.3.5. Nitrogen Adsorption Measurements

The specific surface areas of supports and of MTO modified catalysts were determined via nitrogen adsorption using ASAP 2020 V300 H. The samples were outgassed at 100 °C for 3 h before each measurement. The BET equation was used for calculating the specific surface area of mesoporous materials. The pore size distribution was obtained from the Barrett-Joiner-Halenda correlation.

#### 3.3.6. Ultraviolet-Visible Spectroscopy (UV-Vis)

The structural characterization of samples was performed using UV-Vis spectroscopy. The UV-Vis spectral studies were carried out using the Perkin Elmer Lambda 35 solid state spectrometer in the range of 200–1100 nm with a slit width of 4 nm and a scan speed of 240 nm/min. A Spectralon integration sphere was applied to collect diffuse reflectance spectra of the powder samples. Data evaluation was performed with the UV WinLab (Lambda 35) software tool.

#### 3.3.7. Fourier Transform Infrared Spectroscopy (FTIR)

The structural characterization of samples was performed using the NICOLET 6700 FTIR spectrometer with a Continuum microscope (T, R); measurements were made in the range of 7500–350 cm^−1^. The FTIR spectrometer was equipped with deuterated triglycinesulphate (DTGS) as a detector. Data evaluation was carried out with the OMNIC software tool.

#### 3.3.8. Pyridine Adsorption-Desorption with FTIR

The acidity of the pure supports and of the supports modified by MTO was measured by infrared spectroscopy (ATI Mattson FTIR) using pyridine (≥99.5%) as a probe molecule for qualitative and quantitative determination of both Brønsted and Lewis acid sites. The samples were pressed into thin pellets (10–25 mg) that were pretreated at 450 °C before the measurement. Pyridine was first adsorbed for 30 min at 100 °C and then desorbed by evacuation at different temperatures. Three different temperature ranges were used for the desorption of pyridine; weak, medium and strong sites were defined at 250–350 °C, medium and strong sites were defined at 350–450 °C and pyridine which remained absorbed even after desorption at 450 °C was associated with strong sites. The numbers of Brønsted and Lewis acid sites were calculated from the intensities of the corresponding spectral bands, 1545 cm^−1^ and 1450 cm^−1^ respectively, using the molar extinction parameters previously reported by Emeis [30]. Catalysts weights were taken into account in the calculations.

#### 3.3.9. XPS-Analysis

The photoemission spectra were measured using a ESCAProbeP (Omicron Nanotechnology Ltd., Taunusstein, Germany) with a monochromatized X-ray source operated at 1486.7 eV. The vacuum chamber base pressure was 10^−1^ mbar. A 1100–0 eV survey spectrum was taken for each sample. The step length was 0.5 eV.

### 3.4. Catalytic Experiments

Liquid phase metathesis reactions over heterogenized MTO were carried out in a batch-wise operating glass reactor. In a typical experiment using anhydrous dichloromethane as a solvent (*V*_L_ = 15 mL) the MTO mass was 50 mg bound on 1000 mg of the support. The molar ratio of MTO: substrate (methyl undec-10-enoate, 4-allylanisole or diethyl diallylmalonate) was 1:17. The dodecane internal standard (0.1 mL) was added to the reaction. The prepared mixture was stirred vigorously at 39 °C and under atmospheric pressure for 22 h. The samples were taken at different time intervals and analyzed by the Shimadzu GC 2010 gas chromatograph with flame ionization detector (FID). The products were confirmed by GC-MS.

MTO leaching from the supports in various media was measured for the prepared catalytic systems. The leaching of MTO was tested in dichloromethane (p.a.), anhydrous dichloromethane and water. The leaching tests were performed in a flask (50 mL) at room temperature. The catalyst complex was calculated to contain 5 mg of MTO. Fifteen mL of leaching media was added and the mixture was stirred for 3 h at room temperature. The catalyst was filtered from the mixture and dried at 80 °C overnight and the amount of MTO on the support was determined by XRF analysis.

## 4. Conclusions

The current report focuses on an in-depth detailed characterization of immobilized methyltrioxorhenium (MTO), which gives catalysts with a wide spectra of utilization. A range of mesoporous materials with different SiO_2_/Al_2_O_3_ contents, namely mesoporous alumina (MA), aluminosilicates type Siral and MCM-41, were used as supports for immobilization of MTO. The supports had varying chemical compositions (silicate, aluminosilicates and alumina), surface areas, pore volumes, particle sizes and other properties. Frthermore, some of the supports were modified by zinc chloride in order to obtain supports with higher acidity. The immobilization of MTO was optimized using these supports. The prepared heterogeneous catalysts were studied in depth and characterized using XRF, AAS, XRD, SEM, physical adsorption of N_2_, UV-Vis, IR (FTIR), FTIR using pyridine as a probe molecule and XPS.

The immobilization of MTO was successful using all supports. The highest immobilized amount of MTO was achieved using Siral 40, Siral 20 and MCM-41 as supports, leading to over 14 mg of MTO on 100 mg of support in the case of nominal loading of 15 mg MTO/100 mg of supports.

No apparent changes in crystal morphology were observed after the modification by zinc chloride or after immobilization of MTO on the supports. Particle sizes and agglomerates remained the same.

Elemental maps of immobilized MTO on ZnCl_2_/S40 for chlorine, rhenium and zinc were achieved using EDS. It was concluded that zinc, chlorine and rhenium were located on the support in the same positions and, therefore, MTO is ideally dispersed on the surface of the support. This conclusion is consistent with the results of XRD spectra, where MTO peaks were not observed in either the MTO-modified catalysts or their ZnCl_2_ modified forms, indicating perfect dispersion of MTO particles on the support materials.

UV-Vis spectroscopy determined d^8^–d^8^ interaction of two Re atoms in MTO/support and MTO/ZnCl_2_/support samples. This fact shows the possibility of the formation of dimers of MTO on the surface of the supports.

The structural characterization of binding MTO was performed by infrared spectroscopy using FTIR. The presence of the asymmetric vibration of Re = O is observed in spectra of samples with immobilized MTO (band around 960 cm^−1^).

Concentrations of Brønsted and Lewis acid sites were determined by FTIR using pyridine as a probe molecule. Significant changes in acidity, both Lewis and Brønsted, were observed after modification by ZnCl_2_ (increase of Lewis acidity) and due to immobilization of MTO.

The X-ray photoelectron spectroscopy of MTO modified supports demonstrated that rhenium is present in MTO / support samples in three oxidation states: Re^7+^, Re^6+^ and Re^4+^.

The results showed that MTO immobilized on mesoporous materials (siliceous/aluminous) represent high-quality catalytic systems. The prepared heterogeneous materials exhibit high surface areas and pore volume, Lewis and Brønsted acidity, stability under various conditions, and the possibility to enhance properties by modification of ZnCl_2_. Hence these materials can be used in various organic syntheses. The catalytic activity of the immobilized MTO on various supports was demonstrated in metathesis reactions of model compounds, namely methyl undec-10-enoate, 4-allylanisole and diethyl diallylmalonate. Very high conversions and selectivities to the desired products were achieved using MTO immobilized on the chosen supports. The positive influence of the modification of supports by ZnCl_2_ on the activity and selectivity of the catalysts was shown. MTO/ZnCl_2_/MA 5 was evaluated as the most active metathesis catalyst in all cases. No leaching of MTO from the supports was observed during the metathesis reactions.

## Figures and Tables

**Table 1.** Immobilized amounts of methyltrioxorhenium (MTO) on supports after immobilization by 5 mg and 15 mg of MTO on 100 mg of support (determined by X-ray Fluorescence (XRF) analysis).

Support	Nominal amount 15 mg MTO/100 mg support		Nominal amount 5 mg MTO/100 mg support	

	Immobilized amount (XRF) (mg MTO/100 mg support)	Success of the immobilization (%)	Immobilized amount (XRF) (mg MTO/100 mg support)	Success of the immobilization (%)
MA 5	12.2	81	4.0	80
Siral 10	11.3	75	4.4	88
Siral 20	14.4	96	4.5	90
Siral 30	13.1	87	4.5	90
Siral 30HP	4.9	33	–	–
Siral 40	14.6	97	4.5	90
MCM-41	14.1	94	4.5	90
ZnCl_2_/MA 5	12.0	80	4.5	90
ZnCl_2_/Siral 20	6.4	43	4.1	82
ZnCl_2_/Siral 40	9.9	66	4.1	82

Note: Reaction conditions: anhydrous dichloromethane, 20 h, RT.

**Table 2.** Immobilized amount of MTO on supports after immobilization by 30 mg of MTO on 100 mg of support (determined by XRF analysis).

Support	Nominal amount 30 mg MTO/100 mg support	
	Immobilized amount (XRF) (mg MTO/100 mg support)	Success of the immobilization (%)
Siral 20	29.4	98
Siral 40	28.8	96
MCM-41	27.5	92

Note: Reaction conditions: anhydrous dichloromethane, 20 h, RT.

**Table 3.** Main textural characteristics of supports and of their MTO modified forms (immobilized amount of MTO was 15 mg on 100 mg of supports).

Support	Surface Area (m^2^/g)	Average pore diameter (nm)	Pore Volume (cm^3^/g)	Difference in		
				Surface areas (%)	Average pore diameters (%)	Pore volume (%)
MA 5	325	4.8	0.47	–	–	–
MTO/MA 5	281	4.9	0.48	14	−2	−2
Siral 10	321	7.0	0.68	–	–	–
MTO/S10	279	6.4	0.52	13	9	24
Siral 20	303	8.1	0.73	–	–	–
MTO/S20	282	7.8	0.61	7	4	16
Siral 40	464	7.9	0.97	–	–	–
MTO/S40	423	7.7	0.87	9	3	10
MCM-41	1057	3.6	1.05	–	–	–
MTO/MCM-41	987	3.5	0.95	7	3	10

**Table 4.** Main textural characteristics of native supports and those modified with zinc chloride and MTO (immobilized amount of MTO was 5 mg on 100 mg of supports).

Support	Surface Area (m^2^/g)	Average pore diameter (nm)	Pore Volume (cm^3^/g)	Difference in		
				Surface areas (%)	Average pore diameters (%)	Pore volume (%)
MA 5	325	4.7	0.45	–	–	–
ZnCl_2_/MA 5	261	4.7	0.35	20	0	22
MTO/ZnCl_2_/MA 5	264	4.4	0.34	−1	6	3
Siral 20	303	8.1	0.73	–	–	–
ZnCl_2_/S20	225	8.1	0.59	26	0	19
MTO/ZnCl_2_/S20	216	8.0	0.57	4	1	3
Siral 40	464	7.9	0.97	–	–	–
ZnCl_2_/S40	337	7.8	0.80	27	1	17
MTO/ZnCl_2_/S40	335	7.8	0.76	1	0	5

**Table 5.** Characterization results of the oxidation states of Re in MTO supported catalysts by X-ray photoelectron spectroscopy (5 mg MTO/100 mg support).

Catalysts	Time of exposition in vacuum (h)	Number of oxidation states of Re (%)		
		Re^VII+^	Re^VI+^	Re^IV+^
MTO/ZnCl_2_/MA5	3	36	40	24
	24	44	41	16

MTO/Siral 40	3	53	26	21
	24	26	41	30

**Table 6.** Metathesis reactions of model compounds over homogeneous or heterogenized MTO.

Reaction	Catalyst	Conversion (%)	Selectivity at 15% conversion
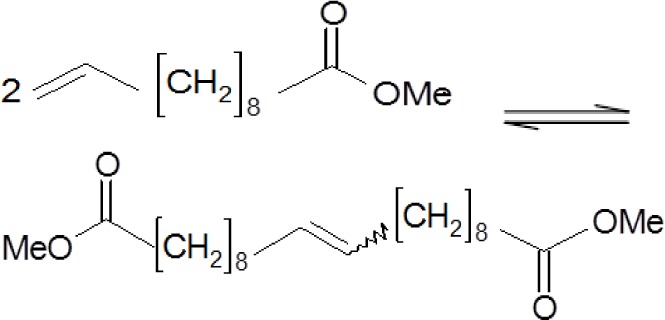	MTO	inactive	–
	MTO/Siral 20	inactive	–
	MTO/ZnCl_2_/S20	49	96
	MTO/Siral 40	57	57
	MTO/ZnCl_2_/S40	80	99
	MTO/MA 5	inactive	–
	MTO/ZnCl_2_/MA5	100	99

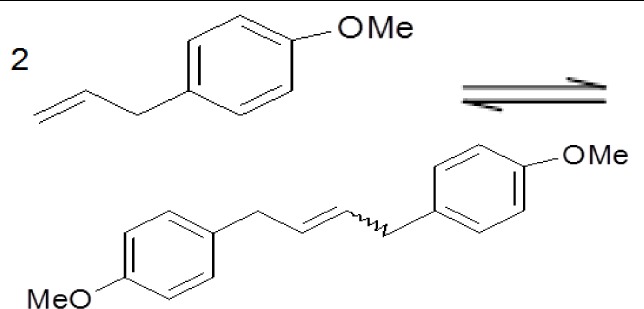	MTO	inactive	–
	MTO/Siral 20	57	94
	MTO/Siral 40	63	95
	MTO/ZnCl_2_/MA5	99	95

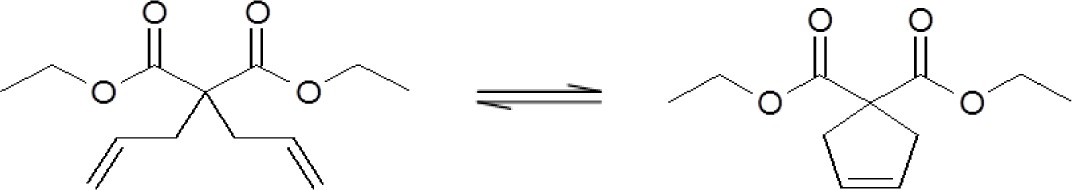	MTO	inactive	–
	MTO/Siral 20	1	100
	MTO/Siral 40	12	100
	MTO/ZnCl_2_/MA5	100	100

Note: Reaction conditions: MTO: substrate 1:17, anhydrous dichloromethane, 39 °C, 22 h.

## Supplementary Materials

Supplementary File 1

## References

[1] Herrmann W.A., Wagner W., Flessner U.N., Volkhardt U., Komber H. (1991). Methyltrioxorhenium as Catalyst for Olefin Metathesis. Angew. Chem. Int. Ed. Engl.

[2] Herrmann W.A., Fischer R.W., Marz D.W. (1991). Methyltrioxorhenium as Catalyst for Olefin Oxidation. Angew. Chem. Int. Ed. Engl.

[3] Herrmann W.A., Wang M. (1991). Methyltrioxorhenium as Catalyst of a Novel Aldehyde Olefination. Angew. Chem. Int. Ed. Engl.

[4] Rudolph J., Reddy K.L., Chiang J.P., Sharpless K.B. (1997). Highly Efficient Epoxidation of Olefins Using Aqueous H_2_O_2_ and Catalytic Methyltrioxorhenium/Pyridine: Pyridine-Mediated Ligand Acceleration. J. Am. Chem. Soc.

[5] Crucianelli M., Saladino R., De Angelis F. (2010). Methyltrioxorhenium Catalysis in Nonconventional Solvents: A Great Catalyst in a Safe Reaction Medium. Chem. Sus.

[6] Herrmann W.A., Fischer R.W., Correia J.D.G. (1994). Multiple bonds between main-group elements and transition metals. Part 133. Methyltrioxorheniumas a catalyst of the Baeyer-Villiger oxidation. J. Mol. Catal.

[7] Murray R.W., Iyana K. (1996). Synthesis of Nitrones Using the Methyltrioxorhenium/Hydrogen Peroxide System. J. Org. Chem.

[8] Zhu Z., Espenson J.H. (1997). Aqueous Catalysis: Methylrhenium Trioxide (MTO) as a Homogeneous Catalyst for the Diels-Alder Reaction. J. Am. Chem. Soc.

[9] Herrmann W.A., Wagner W., Volkhardt U. (1990). Organic Derivatives of Rhenium Oxides and Their Preparation and Use for the Metathesis of Olefins.

[10] Kühn F.E., Herrmann W.A. (2001). Methyltrioxorhenium. Chemtracts.

[11] Jain K.R., Kühn F.E. (2007). Immobilization of organorhenium(VII) oxides. J. Organomet. Chem.

[12] Moses A.W., Raab C., Nelson C.R., Leifeste H.D., Ramsahye N.A., Chattopadhyay S., Eckert J., Chmelka B.F., Scott. S.L. (2007). Spectroscopically Distinct Sites Present in Methyltrioxorhenium Grafted onto Silica-Alumina, and Their Abilities to Initiate Olefin Metathesis. J. Am. Chem. Soc.

[13] Tovar T.M., Stewart S.M., Susannah L.S. (2012). Origin of the ZnCl_2_ Effect on CH_3_ReO_3_/c-Al_2_O_3_ in Olefin Metathesis. Top. Catal.

[14] Di Giuseppe A., Crucianelli M., Passacantando M., Nisi S., Saladino R. (2010). Chitin- and chitosan-anchored methyltrioxorhenium: An innovative approach for selective heterogeneous catalytic epoxidations of olefins. J. Catal.

[15] Jiang J., Zhang Y., Cao D., Jiang P. (2013). Controlled immobilization of methyltrioxorhenium(VII) based on SI-ATRP of 4-vinyl pyridine from halloy site nanotubes for epoxidation of soybean oil. Chem. Eng. J.

[16] Zdenkova R., Leitmannova-Vyskocilova E., Cerveny L. (2012). Methyltrioxorhenium—A catalyst for metathesis reactions. Chem. Listy.

[17] Stekrova M., Vyskocilova E., Kolena J., Cerveny L. (2013). Indene Epoxidation. Chem. Listy.

[18] Buffon R., Auroux A., Lefevre F., Leconte M., Choplin A., Basset J.M., Herrmann W.A. (1992). A surface organometallic approach to the synthesis of rhenium-based catalysts for the metathesis of olefins: CH_3_ReO_3_/Nb_2_O_5_. J. Mol. Catal.

[19] Oikawa T., Masui Y., Tanaka T., Chujo Y., Onaka M. (2007). Lewis acid-modified mesoporous alumina: A new catalyst carrier for methyltrioxorhenium in metathesis of olefins bearing functional groups. J. Organomet. Chem.

[20] Rost A.M.J., Schneider H., Zoller J.P., Herrmann W.A., Kühn F.E. (2005). Methyltrioxorhenium heterogenized on commercially available supporting materials as cyclooctene metathesis catalyst. J. Organomet. Chem.

[21] Buffon R., Choplin A., Leconte M., Basset J.M., Touroude R., Herrmann W.A. (1992). Surface organometallic chemistry of rhenium: Attempts to characterize a surface carbene in metathesis of olefins with the catalyst CH_3_Re0_3_/Nb_2_0_6_. J. Mol. Catal.

[22] Neumann R., Wang T.-J. (1997). Methyltrioxorhenium supported on silica tethered with polyethers as catalystfor the epoxidation of alkenes with hydrogen peroxide. Chem. Commun.

[23] Wang T.-J., Li D.-C., Bai J.-H., Huang M.-Y., Jiang Y.-Y. (1998). Silica Supported Methyltrioxorhenium Complex of γ-(2,2′-Dipyridyl)-aminopropylpolysiloxane as a Novel Catalyst for Epoxidation of Alkenes. J. Macromol. Sci. Pure.

[24] Gago S., Fernandes J.A., Abrantes M., Kühn F.E., Ribeiro-Claro P., Pillinger M., Santos T.M., Gonçalves I.S. (2006). Immobilization of methyltrioxorhenium on functionalized MCM-41. Microporous Mesoporous Mater.

[25] Nunes C.D., Pillinger M., Valente A.A., Gonçalves I.S., Rocha J., Ferreira P., Kühn F.E. (2002). Synthesis and Characterization of Methyltrioxorhenium(VII) Immobilized in Bipyridyl-Functionalized Mesoporous Silica. Eur. J. Inorg. Chem.

[26] Lever A.B.P. (1984). Inorganic Electronic Spectroscopy.

[27] Bek D., Žilková N., Dědeček J., Sedláček J., Balcar H. (2010). SBA-15 Immobilized Ruthenium Carbenes as Catalysts for Ring Closing Metathesis and Ring Opening Metathesis Polymerization. Top. Catal.

[28] Köstlmeier S., Pacchioni G., Herrmann W.A., Rösch N.J. (1996). Structure and properties of dimer, trimer and tetramer aggregates of methyltrioxorhenium (MTO): An *ab initio* study. Organomet. Chem.

[29] Sakthivel A., Raudaschl-Sieber G., Kühn F.E. (2006). Heterogenization of an organorhenium(VII) oxide on a modified mesoporous molecular sieve. Dalton Trans.

[30] Emeis C.A. (1993). Determination of integrated molar extinction coefficients for infrared absorption bands of pyridine adsorbed on solid acid catalysts. J. Catal.

[31] Pillai S.K., Hamoudia S., Belkacemia K. (2013). Metathesis of methyloleate over methyltrioxorhenium supported on ZnCl_2_-promoted mesoporous alumina. Appl. Catal. A.

[32] Kühn F.E., Scherbaum A., Herrmann W.A. (2004). Methyltrioxorhenium and its applications in olefin oxidation, metathesis and aldehyde olefination. J. Organomet. Chem.

[33] Cornils B., Herrmann W.A., Herrmann W.A. (2004). Aqueous-Phase Organometallic Catalysis.

[34] Beck J.S., Vartuli J.C., Roth W.J., Leonowicz M.E., Kresge C.T., Schmitt K.D., Chu C.T.W., Olsen D.H., Sheppard E.W., McCullen S.B. (1992). A new family of mesoporous molecular sieves prepared with liquid crystal templates. J. Am. Chem. Soc.

[35] Xu B., Xiao T., Yan Z., Sun X., Sloan J., González-Cortés S.L., Alshahrani F., Green M.L.H. (2006). Synthesis of mesoporous alumina with highly thermal stability using glucose template in aqueous system. Microporous Mesoporous Mater.

