# Supplementary material for: Immobilization of Methyltrioxorhenium on Mesoporous Aluminosilicate Materials
Source: Materials (Basel). 2014 Mar 28;7(4):2650–68. doi: 10.3390/ma7042650 (PMC5453337; doi:10.3390/ma7042650)

Supplementary Information

Morphological Studies by Scanning Electron Microscopy (SEM)

The morphology of the prepared supports, of their zinc chloride modified form and of the catalytic system with immobilized MTO (15 mg MTO/100 mg of the support and 5 mg MTO/$ZnCl_2$/support in case of using zinc modified support) was studied by scanning electron microscopy (Figures S1–S3).

The support materials were characterized by different particle sizes. Prepared MCM-41 had small particles (<1 µm) which form large agglomerates (Figure S1a). No apparent changes in crystal morphology were observed after immobilization of MTO on MCM-41. Particle size and agglomerates remained the same (Figure S1b).

The prepared mesoporous alumina MA 5 (Figure S2a) is quite heterogeneous in particle size. The smallest particles are smaller than 10 µm and form agglomerates of sizes of about 40 µm. No apparent changes in crystal morphology were observed after modification of the MA support (Figure S2b) and Siral materials (Figure S3) by zinc chloride.

Immobilized MTO was not visible by scanning electron microscopy on pure supports, and the same also applies to their zinc-modified form (Figures S1–S3).

Figure S1. Scanning electron micrograph of (**a**) MCM-41 and of (**b**) MTO/MCM-41.

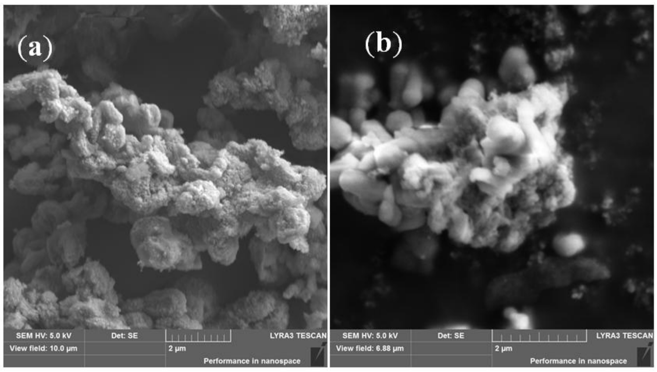

Figure S2. Scanning electron micrograph of (**a**) mesoporous alumina (MA 5); (**b**) MA 5 modified by $ZnCl_2$ and (**c**) MTO/$ZnCl_2$/MA.

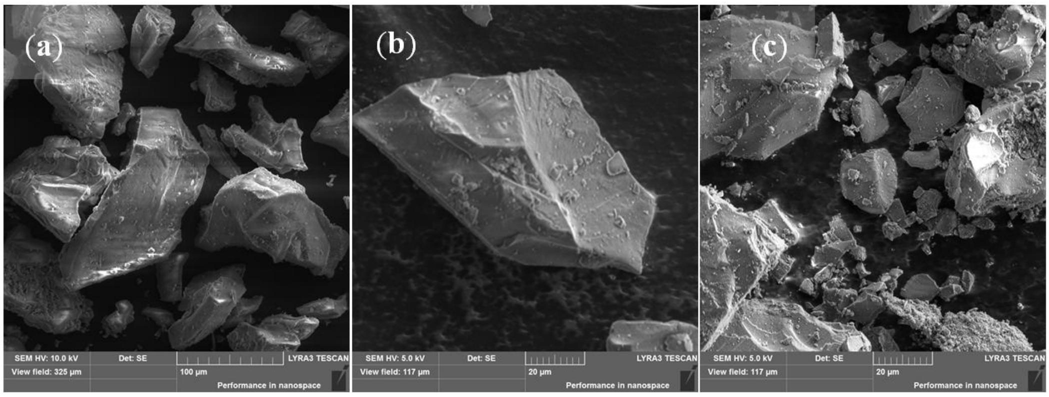

Figure S3. Scanning electron micrograph of (**a**) MTO/Siral 20; (**b**) $MTO/ZnCl_2$/Siral 20; (**c**) MTO/Siral 40 and (d) $MTO/ZnCl_2$/Siral 40.

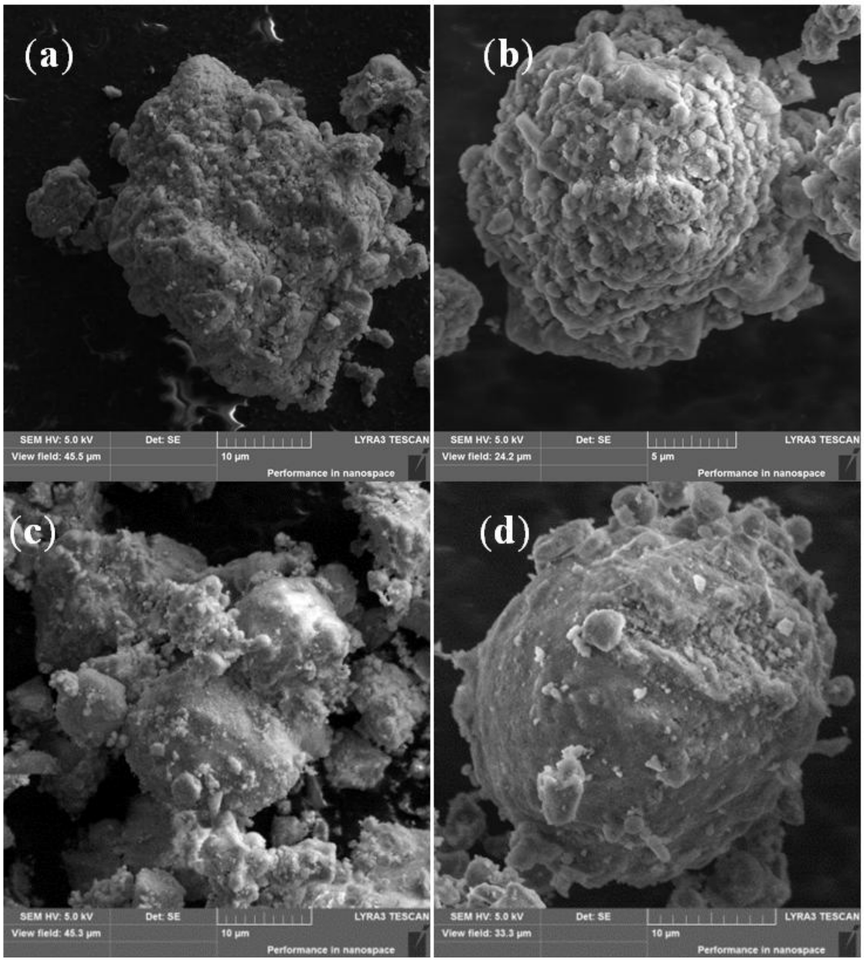

Supplement: Supplementary File 1 [file materials-07-02650-s001.pdf]
